# Current Therapeutic Strategies in BRAF-Mutant Metastatic Colorectal Cancer

**DOI:** 10.3389/fonc.2021.601722

**Published:** 2021-06-23

**Authors:** Elisa Grassi, Jody Corbelli, Giorgio Papiani, Maria Aurelia Barbera, Federica Gazzaneo, Stefano Tamberi

**Affiliations:** Department of Oncology, Infermi’s Hospital, Azienda Unità Sanitaria Locale (AUSL) della Romagna, Faenza, Italy

**Keywords:** colorectal cancer, BRAF mutation, microsatellites instability, targeted therapy, immunotherapy

## Abstract

Around 8–12% of patients with advanced colon rectal cancer (CRC) present with BRAF alterations, in particular V600E mutation, which is associated with right-side, poorly differentiated and mucinous type tumors. The presence of BRAF mutation (BRAF-mt) has been identified as a hallmark of poor prognosis and treatment optimization in this patient subgroup is an important goal. Currently, the standard of care is an aggressive strategy involving triplet chemotherapy and anti-VEGF agents, but new therapeutic approaches are emerging. Very promising results have been obtained with targeted therapy combinations, such as anti-BRAF agents plus anti-EGFR agents. Furthermore, around 60% of BRAF-mt patients show a strong association with high microsatellite instability (MSI-H) and immune checkpoint inhibitors could represent the new standard of care for this subgroup. The focus of this review is to summarize current strategies for BRAF-mt CRC treatment and highlight new therapeutic options.

## Background 

Colorectal cancer (CRC) is a molecularly heterogeneous disease the second most frequent cause of cancer-related death worldwide (1, 2). BRAF mutations in advanced disease are observed in 8–12% of patients, and T1799A transversion in exon 15, which results in a valine amino acid substitution (V600E), is the most frequent alteration. Around 2% of BRAF-mutant (BRAF-mt) cancers have non-V600E *BRAF* mutations, the prognostic and predictive value of which is still not clear (3). BRAF mutation influences cellular growth, proliferation and differentiation mechanisms through an aberrant activation of the MAPK/ERK pathway (4). BRAF is considered a negative prognostic biomarker and patients harboring this mutation have limited response to chemotherapy. The best treatment option appears to be triplet chemotherapy plus anti-VEGF agents, but data are still limited (5). The use of BRAF inhibitors as single agents has been shown to obtain some benefit, but interesting results were recently obtained from a combination of different targeted therapies, in particular BRAF inhibitors plus anti-EGFR agents plus anti-MEK agents. Furthermore, there is a strong association between BRAF V600E mutation and microsatellite instability and immunotherapy could thus represent a new standard of care in this subgroup (6, 7).

The present summarizes the current therapeutic options for BRAF-mt CRC.

## BRAF Pathway and Microsatellite Instability in CRC

BRAF plays an important role in the activation of RAS/RAF/MEK/extracellular signal-regulated kinase (ERK) signaling cascade that drives cellular growth, proliferation and differentiation, and also other key cellular processes such as migration, apoptosis and cellular survival. RAS guanidine triphosphatase (GTPase) activates RAF family proteins (ARAF, BRAF and RAF1), leading to the phosphorylation of MEK1/2 proteins. These last then activate ERKs and the phosphorylation of ERK transcription factors, which play a key role in a variety of cellular activities. BRAF and KRAS mutations are mutually exclusive and BRAF-mt induces an aberrant and inappropriate activation of the MAPK/ERK pathway (3, 4, 8). BRAF-mt CRC is associated with specific clinical-pathological features, and serrated polyps have been recognized as precursor lesions of the disease (9). These tumors are frequently located in the right colon and are more common in elderly females. They metastasize more frequently to the peritoneum and are associated with poorly differentiated and mucinous subtype, a higher frequency of tumor budding and an infiltrative pattern of invasion with an increased risk of lymphovascular invasion and different tumor infiltrating lymphocyte (TIL) grades (10, 11).

Microsatellites are DNA sequences repeated within coding and non-coding regions of the genome. Mismatch repair (MMR) damage results in genetic hypermutability and leads to microsatellite instability (12). Around 3% of MSI-H colon cancer is due to a germline mutation in MMR genes (Hereditary Non-Polyposis Colorectal Cancer or Lynch syndrome), while another 12% of cases depend on a somatic inactivation of MMR genes, in particular, MLH1 promoter region hypermethylation (13). MSI prevalence is higher in early-stage disease (about 15%) than in advanced stage (about 3–5%) (14, 15). There is a strong association between somatic inactivation of MMR genes and BRAF mutation, with a co-presence of 60%, which, however, is not observed in Lynch syndrome (5, 16). This finding is supported by evidence that BRAF-mutated CRC appears to develop from a “serrated pathway” of carcinogenesis often related to extensive DNA methylation of CpG islands. The methylation of MLH1 promoter (a gene of the MMR system) leads to a ‘sporadic’ microsatellite-instable phenotype (16).

BRAF alteration, in particular V600E alteration, is considered an independent negative prognostic factor in the metastatic setting, as seen from the results of a pooled analysis of the CAIRO, CAIRO2, COIN, and FOCUS studies (17). However, there is evidence of a substantial heterogeneity in the outcome of BRAF-mt patients, suggesting the usefulness of a scoring system based on clinical-pathological features to improve patient stratification and and therapeutic strategies (18).

The prognostic role of the BRAF mutation/MSI-H association is still under debate. BRAF-mutant-MSI-H CRCs show similar clinical-pathological characteristics such as old age, female sex, right-side, mucinous features, poor differentiation, high-grade TILs and peritumoral lymphoid reactions (19). However, there is evidence suggesting that BRAF mutations may differ in their impact on MSI and MSS tumors. In particular, BRAF mutations appear to be an independent negative prognosis factor in early-stage MSS CRC, whereas this has not been shown in the MSI population. Furthermore, in the metastatic setting, there is increasing evidence that MSI-tumors, when stratified by BRAF status, do not significantly differ in terms of survival rates (17). No definitive conclusions can be drawn about this issue because of the low frequency of both MSI and BRAF mutations, indicating the need for further data. Finally, BRAF mutations occur outside of codon 600 (non-V600 BRAF mutations) in around 2.2% of cases. Jones et al. reported that these alterations define a clinically distinct subtype of CRC with an excellent prognosis, demonstrating that BRAF-mutant patients are a mixed population in which a tailored approach is needed (20).

## State-Of-The-Art of Therapeutic Strategies in BRAF-MT and MSI-H CRC

### Chemotherapy, Anti-Angiogenic Agents and Anti-EGFR Agents

Given the poor progression-free survival (PFS) and survival (OS) rates of BRAF-mt metastatic CRC, more aggressive therapeutic strategies have been tested in this setting. In a phase II study conducted by Loupakis et al., a triplet regimen of 5-fluroruracil plus oxaliplatin plus irinotecan plus bevacizumab used in a small group of BRAF-mt patients showed encouraging results, with a median PFS of 11.8 months and a median OS of 24.1 months (21). The benefit of FOLFOXIRI plus bevacizumab *vs*. FOLFIRI (5-fluroruracil plus irinotecan) plus bevacizumab as first-line treatment was evaluated in the subsequent phase III TRIBE study (5). The BRAF-mt subgroup (28 patients) showed a positive, albeit not significant, trend in terms of OS (10.7 months *vs.* 19 months, HR 0.84) and PFS (5.5 months *vs.* 7.5 months, HR 0.57) with respect to the control arm (5). However, this intensive approach was only used in a small number of patients and was limited by a higher rate of toxicity.

On the basis of preclinical data and given the role of BRAF in RAS/RAF/MEK/ERK pathway, it has been hypothesized that BRAF mutation may be an indicator of resistance to monoclonal antibodies targeting EGFR. However, the predictive role of this alteration has yet to be confirmed (22). Retrospective data are insufficient to draw any definitive conclusions about the role of BRAF mutations in determining primary resistance to anti-EGFR agents. To date, the PICCOLO trial is the only study to have reported a deleterious effect of adding panitumumab to chemotherapy in BRAF-mt patients (23).

Two meta-analyses have been carried out on the role of BRAF mutations in predicting response to anti-EGFR agents. Pietrantonio et al. analyzed 10 clinical trials for a total of 462 BRAF-mt patients, concluding that cetuximab or panitumumab did not improve PFS (HR 0.88; p = 0.33) or OS (HR 0.91; p = 0.63) compared to standard chemotherapy or best supportive care (24).

In another meta-analysis of eight studies (351 BRAF-mt patients), excluding the NORDIC and FIRE-3 trials, the authors reported a non significant interaction between anti-EGFR treatment and BRAF mutations, concluding that the data were insufficient to justify the exclusion of anti-EGFR agents for the treatment of BRAF-mt cancer (25).

Finally, the FIRE-3 trial compared the association of FOLFIRI plus bevacizumab with FOLFIRI plus cetuximab for the first-line treatment of patients with RAS wild-type disease. In the subgroup of BRAF-mt patients (about 14%), FOLFIRI-cetuximab obtained a higher overall response rate (ORR) than FOLFIRI-bevacizumab without, however, any differences in PFS and OS (26). These data suggest that anti-EGFR agents plus chemotherapy do not significantly improve the outcome of BRAF-mt patient with respect to VEGF inhibitors in a first-line setting, with the exception of the response rate.

### BRAF Inhibitors and Targeted Treatment Combinations

BRAF inhibitors such as vemurafenib or encorafenib (tyrosine kinase inhibitors specific to the ATP-binding domain of BRAF V600E) have been tested in patients with CRC or melanoma with BRAF V600E mutations. Kopetz et al. observed clinical activity of BRAF-inhibitor therapy in 21 pre-treated patients with V600E BRAF-mt CRC, reporting a partial response in 14 patients lasting 21 weeks and stable disease in seven patients lasting >8 weeks (27). Conversely, no responses were observed in the 10 patients with metastatic CRC enrolled in the basket trial MO28072. Median PFS and OS were 4.5 and 9.3 months, respectively (28). A possible reason for this resistance was seen in pre-clinical studies in which cell lines with BRAF inhibition showed a feedback activation of EGFR, which is highly expressed in colon cancer cells (29, 30). Some authors have also hypothesized that the activation of the PI3K/AKT pathway may explain the resistance to BRAF inhibitors in BRAF-mt CRC cells (31).

This suggests that better results could be obtained by combining BRAF inhibitors with other targeted agents such as anti-EGFR agents and/or PI3K inhibitors. The association of vemurafenib and cetuximab was evaluated in 27 patients with BRAF-mt metastatic CRC (mCRC) in Hyman et al.’s basket trial, the authors reporting one objective response and a median PFS and OS of 3.7 and 7.1 months, respectively (28). Similarly, in a pilot study of 15 patients with metastatic CRC in progression of at least one treatment, panitumumab plus vemurafenib showed modest efficacy, with tumor regression observed in 10 out of 12 cases (32). Given the interesting results obtained in melanoma, Corcoran et al. conducted a study on 43 patients with BRAF V600-mt CRC treated with dabrafenib (a BRAF inhibitor) plus trametinib (an anti-MEK agent). About 12% of patients achieved a partial response, while 24 (56%) patients achieved stable disease (30).

A combination of triplet targeted therapies was tested to overcome the most important mechanisms of BRAF inhibitor resistance such as EGFR over-activation and PI3K pathway modulation (33). Van Cutsem et al. carried out a phase I/II trial (MEK116833) in which 35 patients received dabrafenib plus trametinib plus panitumumab. The ORR was 21% and median PFS was 4.1 months, but patients experienced significant skin toxicity (33). A phase 1b trial evaluated the therapeutic effect of the combination of encorafenib and cetuximab ± alpelisib in 28 patients, the authors reporting an ORR of 32.1% in the triplet arm compared to 23.1% in the dual arm. PFS was 4.3 months in the triplet therapy group and 3.7 months in the dual treatment arm. The most common toxicities observed for the triplet treatment were hyperglycaemia (11%) and increased lipase (7%) (34). More research is warranted to evaluate the benefit of adding alpelisib to the encorafenib–cetuximab combination.

Interesting results have also been obtained from a combination of BRAF inhibitors with anti-EGFR agents and chemotherapy. The SWOG S1406 study randomized 99 patients with BRAF-mt pre-treated CRC to receive irinotecan plus cetuximab ± vemurafenib. Median PFS was 4.4 months in the triplet arm compared to 2.0 months in the doublet arm (HR 0.42; p = 0.0002). The authors reported a higher ORR in the triplet combination (16% versus 4%, p = 0.09) (35).

The results of the BEACON trial were recently published. In this phase 3 trial, 665 patients with BRAF V600E-mt metastatic CRC in progression after one or two previous regimens were randomized to receive encorafenib, binimetinib and cetuximab or encorafenib and cetuximab or the investigator’s choice of therapy. The triplet arm showed a significantly longer OS (9.0 months *vs.* 5.4 months, HR 0.52, p <0.001) and a higher response rate than the standard arm (26% *vs.* 2%, p <0.001). Around 58% of patients assigned to the triplet therapy experienced grade 3 or more adverse events compared to 50 and 61% in the doublet-therapy and control groups, respectively (36). A recent update of the BEACON study showed no difference in OS between the triplet arm and the doublet arm (9.3 *vs.* 9.3 months). On the basis of these results, the European Medicines Agency (EMA) approved the association of encorafenib and cetuximab for the treatment of patients with BRAF V600E-mt mCRC who have already undergone one or two lines of chemotherapy for metastatic disease.

### Immune Checkpoint Inhibitors

BRAF gene mutation is closely associated with high MSI through its relationship with high-level CpG island methylator phenotype (CIMP) and MLH1 promoter methylation. Around 52% of MSI tumors also have BRAF mutations, and 55% of BRAF-mt tumors show MSI (16). Immunotherapy, in particular, immune checkpoint inhibitors (antibodies directed against programmed cell death protein 1, PD1, or its ligand, PDL1), have positively impacted the treatment of several tumors (37, 38). However, studies evaluating immunotherapy in CRC failed to demonstrate a benefit, with the exception of patients with MSI-H tumors (39). In the CheckMate 142 trial, 119 pre-treated MSI patients, of whom 24% were BRAF-mt, received nivolumab plus ipilimumab. ORR was 55% and the disease control rate for ≥12 weeks was 80%. The treatment showed a manageable safety profile (40).

Nivolumab plus ipilimumab is currently approved for patients with MSI-H/dMMR CRC after progression on prior treatment with a fluoropyrimidine, oxaliplatin, and irinotecan.

The results of the phase II KEYNOTE-164 trial on the antitumor activity of pembrolizumab in previously treated, metastatic, MSH-H/mismatch repair-deficient (MSI-H/dMMR) CRC were recently published. The subgroup of 61 patients that had undergone >2 prior lines of standard therapy (cohort A) showed an ORR of 33%, a median PFS of 2.3 months and a median OS of 31.4 months at median follow-up of 31.3 months, while those who had received >1 prior line of therapy (cohort B) had an ORR similar to that of cohort A and a median PFS of 4.1 months. The median OS was not reached at a median follow-up of 24.2 months. Treatment showed a well-tolerated safety profile (7).

Finally, the results of a phase III trial comparing the use of first-line pembrolizumab with chemotherapy in MSI-H advanced CRC were recently published. The chemotherapy arm showed a median PFS of 8.2 months (HR 0.60, p = 0.0002), while the group treated with immunotherapy had a median PFS of 16.5 months, thus representing a new standard of care for the first-line treatment of MSI-H colorectal cancer (41). Furthermore, preliminary data reported by Frederick et al. suggest a potential synergy between BRAF-targeted therapy and immunotherapy, and this combination could be an interesting option to evaluate in MSI-BRAF-mt CRC (42).

**Figure 1:**
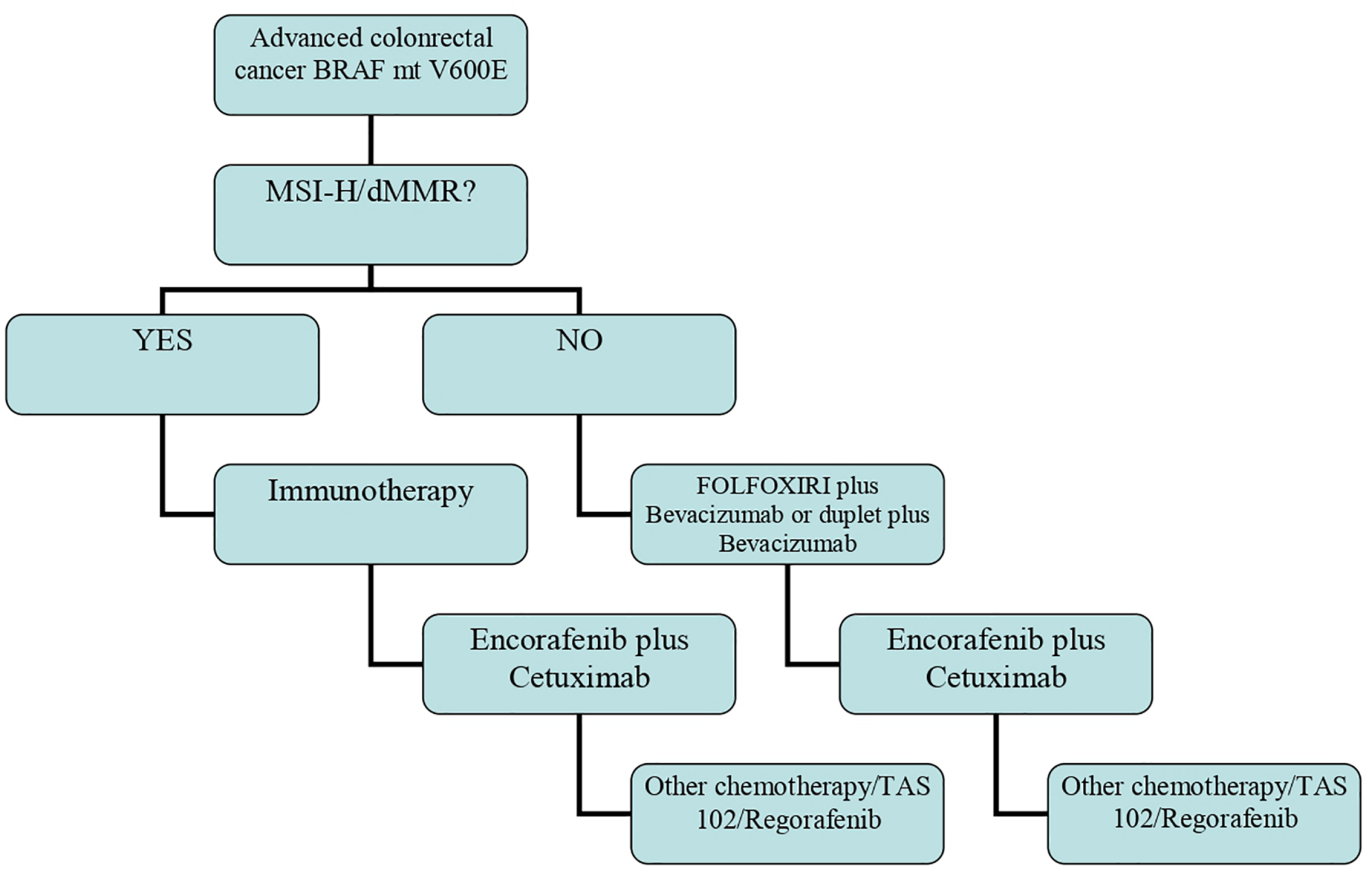
Algorithm of therapy in BRAF mt advanced colon cancer.

The above findings highlight that the algorithm of therapy in BRAF-mt patients is changing and that a different approach must be used for those with MSI-H/dMMR tumors (**Figure 1**). 

## Conclusion 

Therapeutic options for BRAF-mt patients have changed from aggressive chemotherapeutic schedules to targeted treatment combinations and immunotherapy for the MSI-H subgroup. The next step will be to identify the best care strategy and how to personalize the approach, taking into consideration that patients with BRAF-mt CRC are not a homogeneous population.

## Author Contributions

EG, JC, GP, MB, and FG wrote the manuscript. ST supervised the project. All authors contributed to the article and approved the submitted version.

## Conflict of Interest

The authors declare that the research was conducted in the absence of any commercial or financial relationships that could be construed as a potential conflict of interest.
